# Perceiving inter-leg speed differences while walking on a split-belt treadmill

**DOI:** 10.1038/s41598-024-85091-8

**Published:** 2025-01-08

**Authors:** Carl Müller, Karl Kopiske

**Affiliations:** Cognitive Systems Lab, Institute of Physics, Chemnitz University of Technology, Reichenhainer Str. 70, 09126 Chemnitz, Germany

**Keywords:** Self-motion, Perception and action, Just-noticeable differences, Walking, Sensorimotor adaptation, Human behaviour, Sensorimotor processing

## Abstract

Walking is one of the most common forms of self-motion in humans. Most humans can walk effortlessly over flat uniform terrain, but also a variety of more challenging surfaces, as they adjust their gait to the demands of the terrain. In this, they rely in part on the perception of their own gait and of when it needs to be adjusted. Here, we investigated how well N = 48 participants detected speed differences between two belts of a split-belt treadmill. As participants walked at a constant speed, we either accelerated or decelerated one of the belts at quasi-random intervals and asked participants to judge their relative speeds in a two-alternative forced-choice task. Using an adaptive psychophysical procedure, we obtained precise perception-threshold estimates for inter-leg speed differences after accelerating or decelerating one belt. We found that most participants could detect even very small speed differences, with mean threshold estimates of just over 7% for both perturbation types. These were relatively stable within, but highly variable across participants. Increased-speed and decreased-speed thresholds were highly correlated, indicating that despite different biomechanics, the detection mechanisms might be similar. This sheds light on how perceiving their own motion helps humans manage interlimb coordination in perturbed walking.

## Introduction

Walking is one of the most universal forms of locomotion for humans. In order to ensure a safe gait, humans have to continuously adjust their movements to changes in the environment^1^ to deal with flat and uniform terrain, as well as slippery surfaces^2^ and obstacles^3^, taking into account both their environment and their own self-motion. This process of continuous recalibration is common to most motor actions, but especially important in walking, as gait instability caused by disturbances like slips or stumbles can lead to falls, which are highly associated with fractures or serious injuries^4,5^.

A well-established way to measure motor adjustments is experimentally introducing perturbations and observing the motor output, or physiological responses such as EMG^6^. For example, participants may adapt their gait by adjusting kinematics like step length, double-support time or stride-length, the latter in particular being a popular measure of gait adaptation in real-world walking situations, when walking also curves than straight lines^6–9^. Further, gait may be also adapted by changes in kinetics like joint angles, limb positions or the muscular outputs resulting for instance in changes in the ground reaction forces^10,11^. It has been shown that such sensorimotor adjustments can occur both with and without the actor detecting the perturbations, that is, *explicitly* and *implicitly*^12^. Sensorimotor learning^13^ combines these explicit and implicit adaptation processes^14^, with a broad involvement of explicit strategies in adaptation^15^. In walking, relations between detecting perturbations and adaptation were shown by Hoogkamer et al. (2015), as participants with a lower perception threshold walked with less asymmetry in stance time but more asymmetry in limb excursion in response to split-belt speed perturbations. From these results follows the hypothesis that the awareness of perturbations might play a key role in recalibrating sensorimotor actions to prevent falls and injuries. To clarify under what conditions participants consciously perceive the perturbation of their gait and thus could apply explicit strategies for adapting their gait, it is important to determine how well humans can detect typical perturbations of self-motion.

Psychophysical investigations of detecting perturbations in walking often measure the perception threshold of inter-leg speed differences by introducing split-belt perturbations^8,16–18^. That is, experimenters will present different belt speeds for each leg, ask participants to judge the relative speeds of the belts, and measure the just-noticeable differences (JNDs) for participants perceiving the motion of the belts and their own perturbed walking apparatus. Typically, participants walk on a split-belt treadmill, while speed is perturbed for a period of time ranging from only a single stance phase^18^ or one full stride cycle^17^, to multiple steps^9^ and even up to 2 min walking^16^. Perturbation duration mainly depends on methods such as the perception threshold paradigm^16,19^, increasing perturbation each second up to a defined maximum speed difference, discrimination tasks^9,18^ mostly with short perturbation times and a self-selected walking speed or by using a two-alternative forced-choice (2AFC) task^17^, with defined speed perturbations for each participant over a full stride cycle. Mean JNDs of split-belt speed perturbations obtained from these paradigms range from < 9%^17^ up to 13%^16^ for young and healthy participants, but vary depending on these different procedures, psychophysical methods and experimental setup. Further, the number of trials combined with small sample sizes and often relatively few trials near the perception threshold, means that estimates of JNDs for split-belt speed perturbations from individual studies can be less precise and reliable than we would wish, and more importantly, certain parameters such as inter-individual variability cannot be sensibly estimated at all.

In most recent studies, experimenters looked exclusively at the detection of treadmill belt *acceleration* perturbations^16^. Less in known about detection performance if one belt is suddenly *decelerated*. While mismatches in many classic psychophysics like visual motion perception are symmetrical in that making one stimulus less intense can be considered equivalent to making another more intense^20^, this is not the case in self-motion perception, which is typically supported by a variety of signals that may respond differently to externally induced changes, and specifically in walking. Biomechanically, a deceleration leading to stumbling will lead to the braking force compressing joints and limbs while placing the foot, rather than stretching these as happens while accelerating, thus further leading to different receptors being used, respectively^21,22^. These fundamental differences might also result in changes in the JNDs. Thus, it is important to determine the precise perception threshold (JND) for each perturbation type individually to investigate distinct properties leading to a better detection performance, which motivates our main questions: (i) how well can participants detect speed differences between legs, (ii) how large the variability in this between participants is, and (iii) if there are differences in the JNDs and variability between perturbations with *increased* and *decreased* speed. Using a large sample, we investigated the perception thresholds (JNDs) of speed-increase and speed-decrease perturbations while split-belt walking. To do this, we changed the speed of the perturbed belt during swing phase so that participants were exposed to sudden speed differences between belts. As a methodological improvement, perturbations were introduced using an adaptive method, the QUEST procedure^23^, which provides an on-the-fly estimate of the threshold after each trial during the experiment based on the previous responses in the 2AFC task, thus characterizing participants’ perturbation detection. We also quantify the similarity between perturbation types, which had previously been hard to determine.

## Methods

### Participants

A total of N = 52 participants were recruited via a TU-Chemnitz online mailing list. Participants were eligible if they had no neurological or walking impairments and a body mass of less than 130 kg (the latter due to technical constrains of the setup). Four participants had to be excluded from analysis due to technical issues. This left us with an eventual sample of N = 48. No power analysis was conducted since our main goal was not hypothesis testing but parameter estimation and variability was difficult to estimate from previous studies. The analyzed sample included 35 women and 13 men with an average age of 22.0 years (between 19 and 37), average height of 171.5 cm ± a standard deviation of 8.5 cm, average body mass 65 kg ± 11 kg and average leg length of 92.4 cm ± 5.0 cm. These measurements were used for motion tracking and collected after participants reported being sufficiently rested and focused to complete the experiment in a questionnaire prior to the experiment. Participants were naïve to the experimental hypotheses. After participation, participants were debriefed and received either course credit or a monetary reimbursement of 10€/h. All experimental procedures were in accordance with the 2013 Declaration of Helsinki as well as approved by the appropriate body (Chemnitz University of Technology ethics committee, reference no. 101628179) and participant data were protected according to institutional regulations.

### Setup and procedure

**Fig. 1:**
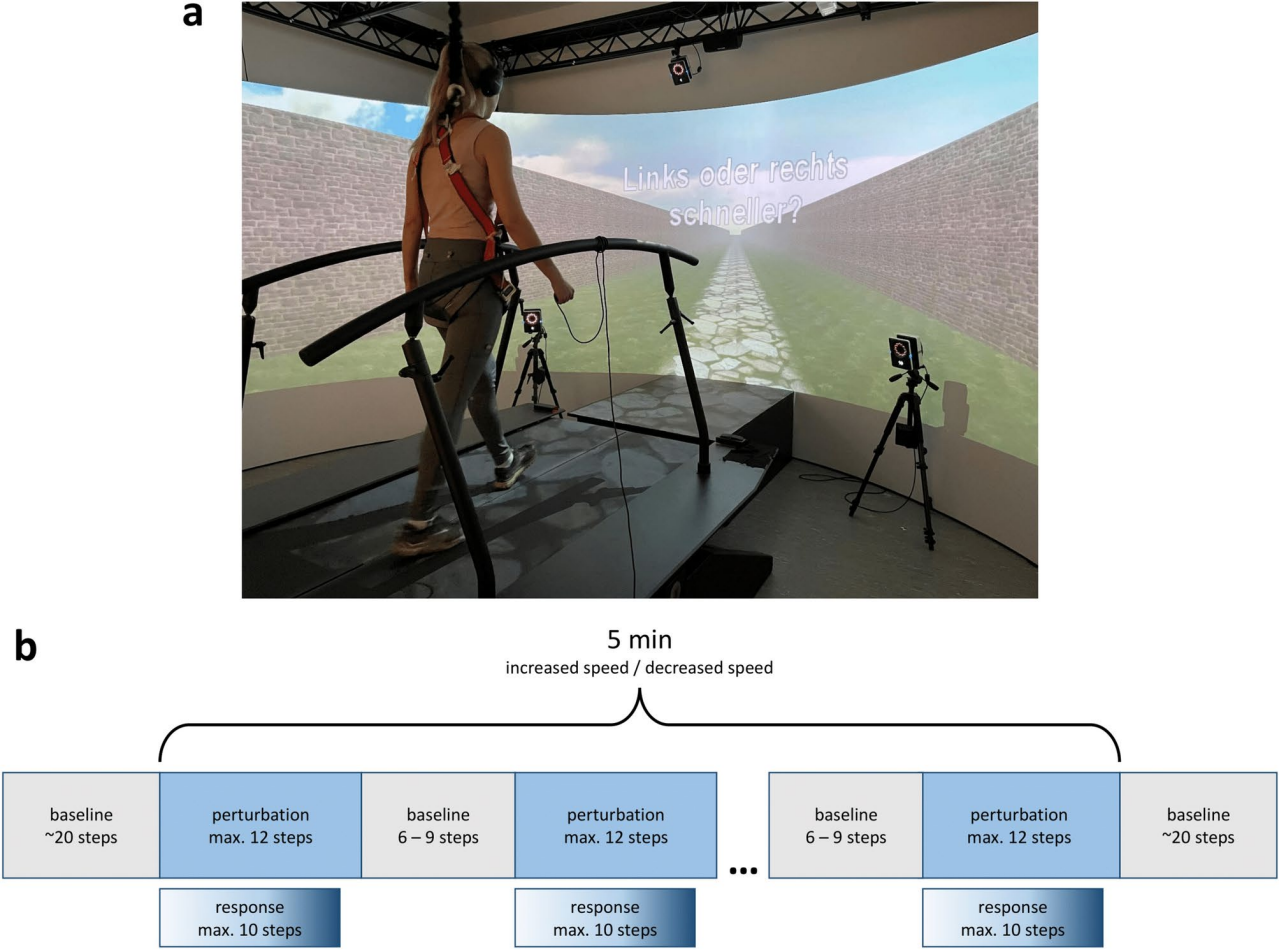
Experimental setup and procedure. (**a**): Virtual environment with a participant walking on the split-belt treadmill along an endless road scene, secured with a safety harness while holding the response controller. Infrared cameras around the treadmill recorded marker positions. The question used for the 2AFC task was displayed on the screen (German: „Links oder rechts schneller? “, translating to “left or right faster?”), indicating participants to give a response. (**b**): Procedure of the experimental blocks, each starting with a baseline phase, then altering between perturbations (block-wise either speed increases or decreases) and short baseline periods for 5 min and ending with baseline walking. The number of perturbed steps depended on the response timing with a maximum of 10 steps, baseline steps were randomized between 6 to 9 steps between perturbation trials.

Participants walked on a split-belt treadmill in a GRAIL system allowing high-precision real-time motion capture in front of a curved 240° projection screen (Fig. 1a). The visual environment was a naturalistically simulated endless road scene with lateral walls, projected 2.5 m in front of the participant at 60 Hz enhanced by a floor projection on the treadmill. Each belt could be accelerated separately with minimal delay^24^ to induce speed perturbations, which were triggered using ground-reaction forces (GRFs) recorded at 250 Hz by force plates below each belt.

For motion capture, we used the Vicon Plug-In Gait lower-body model (Vicon Motion Systems, Yarnton, UK) with 16 retro-reflective markers placed on participants’ body segments. This preparation was always done by the same experimenter to increase reliability^25^. The exact three-dimensional position of the markers was recorded at 250 Hz by 10 infrared cameras at different positions around the treadmill.

Prior the experiment, we measured participants’ biometric dimensions necessary for the gait model (including height and leg length), applied markers, and calibrated the gait model using a standard procedure (consisting of a T-pose and 5 s of walking). Each experiment started with two training sessions of 1 min perturbed walking, one each for speed being increased on the perturbed belt and one for it being decreased. These were followed by 4 experimental blocks, each with a duration of 5 min perturbed walking and again each consisting of either increased speed on the perturbed belt or speeds being decreased. Walking started by accelerating both belts from 0 m/s to 1 m/s baseline speed in 5 steps at 0.2 m/s, followed by approximately 15 s of baseline walking before the experimenter manually started the first perturbation period (Fig. 1b). A fixed baseline speed was chosen for several reasons: First, keeping the speed constant both within and between participants allows to compare speed differences and perceptual thresholds. Further, using a fixed and somewhat slower speed decreases the risk of too strong perturbations that might lead to falls or injuries and has been shown to not strongly affect the resulting thresholds^16,19^. Motor perturbations were speed differences between the right and the left belt, lasting a maximum of 12 steps on constant speed, with one belt being accelerated or decelerated during first swing phase of each perturbation period and the other continuing to run at baseline speed. We used acceleration and deceleration rates of 3 $m/{s}^{2}$ and -3 $m/{s}^{2}$, respectively, which was sufficient for the belt to reach the target speed during swing phase so that participants experienced the new speed, but not the acceleration or deceleration. The perturbed side was randomly chosen for each perturbation of up to 12 steps, and the magnitude of the perturbation (e.g. the speed difference from baseline) was calculated for each perturbation period by using a QUEST procedure (see section “Stimuli and manipulations”). In a 2AFC task, participants had to respond via button press whether the left or the right belt was running faster. Three steps after perturbation onset, the question (German: „Links oder rechts schneller? “, translating to “left or right faster?”) was displayed on the screen. Participants had a maximum of 10 steps within each perturbation period to give their response by pressing either the left or the right button on a handheld controller but were not instructed to respond as quickly as possible. After giving their response or reaching the maximum step count (in which case the response was counted as a wrong answer and a message was briefly displayed on the screen to respond more quickly the next trial: “Bitte etwas schneller antworten!”, German for “Respond a bit more quickly, please!”), the perturbed belt returned to back to baseline speed after two more steps, and after another 6 - 9 steps (randomized for each perturbation), the next perturbation started. While walking, participants wore noise-cancelling headphones to prevent them from using auditory cues to which belt was changing speed.

### Stimuli and manipulations

The main manipulation were speed differences between the left and the right belt of the treadmill, to be judged by the participants. These judgements were used to estimate the just-noticeable speed differences and adjust the strength of the next perturbation accordingly, using a QUEST procedure^23^, an adaptive psychophysical method for threshold measurement, where the best threshold estimate is updated on each trial and presented on the next trial. This method is often used for estimating various sensory thresholds as it increases trials near individual thresholds, resulting in higher reliability compared to many other threshold measurements^26,27^. For the first threshold estimation and thus strength of the first perturbation, we used a rather conservative estimate based on previous findings in other split-belt setups^16–18^ of 10% difference between belts, that is, 0.1 m/s. We set the parameters of the QUEST to account for the properties of our design: the standard deviation was set to the relatively large value of 0.4 m/s, to account for the fact that we used two different perturbation types (speed increased and decreased). We set the other parameters to β = 3.5 and δ = 0.01, typical values suggested by Watson and Pelli^23^. Thus, each block started with a perturbation of 1 m/s ± 0.1 m/s and all following perturbation magnitudes were calculated using the QUEST. We did not use a termination criterion but instead used blocks of 5 min of perturbed walking, which was comfortable for all participants and avoided fatigue. Thus, number of trials (i.e. perturbations) within the QUEST varied between blocks for each participant. The last JND estimate of each block (which, in a QUEST procedure, represents the current best threshold estimate that is updated after each trial and uses the information of all collected trials) was taken as the threshold estimate for the corresponding participant and block.

### Data processing and analyses

Kinematic data from motion capture was processed by applying a cubic-spline interpolation and a Savitzky-Golay Filter^28^ with a window of 124 ms to all relevant markers. The mean proportion of missing data was 0.7%. For step detection, we measured ground-reaction forces (GRFs), calculated the combined forces of both belts, and used the maxima and turning points of the signal filtered with a width of 524 ms (chosen to cover one, but never two steps) to detect steps robustly offline. These measurements were used to analyze *steps to response* and to investigate biomechanical adaptation processes.

Main analyses addressed the threshold estimation of speed perturbations while split-belt walking. For each participant, we received four final threshold estimates, that is, one per block, with two blocks for each of the two perturbation types. We then averaged the JND estimates of the same perturbation type of one participant and compared these JNDs of increased and decreased speeds over all participants using a paired t-test. Further, we calculated 5th and 95th percentiles for JNDs to assess between-participant variability for each perturbation type. Using the block-wise JNDs, we then compared the final JNDs for the two blocks of the same perturbation type by using a paired t-test and further calculated the correlation between block-wise measures within each type to quantify reliability of the JND measurement. These correlations were compared to the correlation between estimates of different types. Considering the relevance of null differences, we additionally calculated Bayes factors corresponding to all t-tests^29^, using a medium-width prior (r = 0.707 as used by Morey & Rouder, 2018, ^30^), as well as for all correlation analyses (with a medium-width prior of r = 0.333). As additional analyses, we looked at how quickly participants converged to the thresholds, and addressed the question if later responses were aligned with smaller JNDs by correlating the mean *steps to response* and the JNDs separately for the two perturbation types. This could also shed light on possible response strategies, such as that some participants—but not others—deliberately take more time to make the right decision when they are unsure. Data and analysis scripts are available at https://doi.org/10.17605/OSF.IO/B7K82.

## Results

### Perception thresholds and variability

**Fig. 2:**
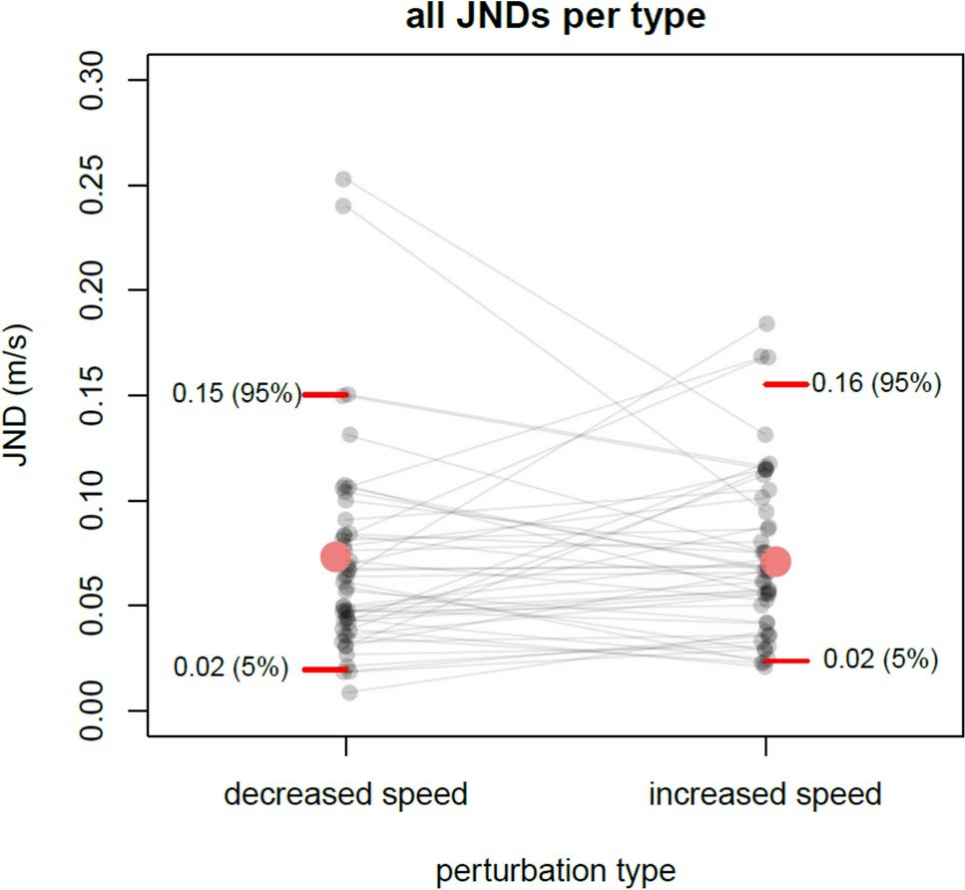
Mean JNDs per participant for increased and decreased speeds. Black semi-transparent dots show the individual data, the larger red dots the overall mean. Inline descriptions at the red lines indicate the 5% and the 95% quantiles, respectively. Shaded lines connecting threshold of the same participant.

We analyzed the detection performance in each perturbation type over all participants and found slightly smaller mean JNDs for decreased-speed perturbations (JND = 0.071 ± a standard deviation of 0.05 m/s) compared to increased-speed perturbations (JND = 0.073 ± 0.04 m/s) but with no statistical difference (*t*(47) = 0.34, p = 0.736), also confirmed by the corresponding Bayesian t-test BF_10_ = 0.17. As is standard procedure, non-responses were counted as wrong answers. Their proportion was 2.6%. As these JNDs are relative to the baseline speed of 1 m/s, differences in speed can be directly viewed as percentage differences of 7.1% (speed increased) and 7.3% (speed decreased). Individual JNDs per participant for each perturbation type are shown in Fig. 2.

The mean threshold estimates are in the range with typical findings in literature. However, Fig. 2 shows that individual thresholds of some participants were much lower than those means. Making use of our large sample, we looked at the differences between the 5% and the 95% percentiles of JNDs. These were different by a factor of 6.6 for increased speeds, and a factor of 7.7 for decreased speeds. These factors indicate huge inter-individual differences in JNDs between the percentiles and within each perturbation type, similarly so for both types, reaching from 2% up to 15% for decreased speeds and up to a JND of 16% for increased speeds at the 95% percentile.

### Stability of the JND measurement

**Fig. 3:**
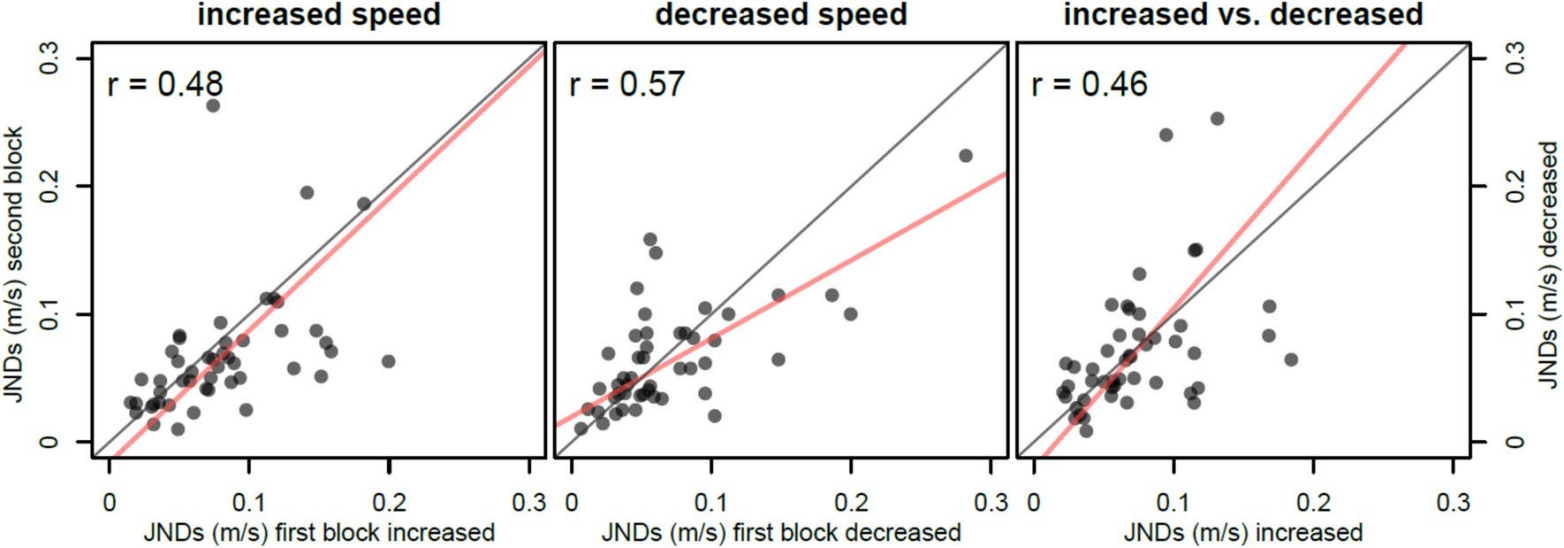
Correlations of mean JNDs for first and second occurred blocks of increased-speed perturbations (panel 1) and decreased-speed perturbations (panel 2) as well as for overall increase and decrease blocks (panel 3). Each dot represents one participant, the red lines indicate the Deming corrected regression line. Solid black line indicates unity.

Next, we tested reliability in the mean JNDs of the two blocks for the same perturbation type. We found descriptively larger JNDs for the first blocks of each type (Table 1), but no statistically significant differences in JNDs compared to the second block, neither for increased speeds (*t*(47) = 1.96,* p* = 0.057) with a corresponding Bayes factor of BF_10_ = 0.90, nor for decreased speeds (*t*(47) = 1.18,* p* = 0.245) with BF_10_ = 0.30, and again relatively large variability in the mean threshold estimates. The correlations between the first and second block were comparable for the two types of perturbation (increase-blocks: r = 0.48, Fig. 3, panel 1; decrease-blocks: r = 0.57, Fig. 3, panel 2), indicating high reliability.

**Table 1:** Mean JNDs per block and perturbation type with standard deviation.

JND	increased speed (± sd)	decreased speed (± sd)
first block	7.99 ± 4.5%	7.57 ± 6.9%
second block	6.66 ± 4.7%	6.61 ± 4.2%

First and second block refers to the first and second block of each perturbation type, respectively, as each participant completed two blocks with increased-speed perturbations and two blocks with decreased-speed perturbations. Displayed are arithmetic means across participants and the corresponding standard deviations.

Being able to calculate reliabilities for each perturbation type also allowed us to compare these to the correlation between increased-speed perturbations and decreased-speed perturbations. This was of particular interest, as both types provide some shared, but also some different sources of information (e.g. biomechanical cues) to detect differences – thus, it is not clear a priori whether detecting increases and decreases in speed would be based on the same cues. Across participants, we found a correlation of the mean JNDs for increases and decreases of *r* = 0.46 (Fig. 3, panel 3). This does not differ significantly from the theoretical upper bound of a correlation of two imperfectly measured variables given their reliabilities, which is given by

1 $${r}_{y1,y2 max}=\sqrt{{r}_{y1,y1}*{r}_{y2,y2}}$$

^31^. Here, the observed correlation of r = 0.46 was not significantly different from ${r}_{max}=\sqrt{0.48*0.57}=0.52$ with *t*(46) = − 0.44, *p* = 0.662. A Bayesian analysis using the 95% confidence interval of *r*_*max*_ as the null-interval gave us a BF_01_ = 20.48 for the correlation being within this interval vs. it being outside of it, that is, strong evidence against a difference between *r* and *r*_*max*_. The vast majority of variability in the JNDs was thus between participants rather than between the different perturbation types.

### Effects of timing and responses

**Fig. 4:**
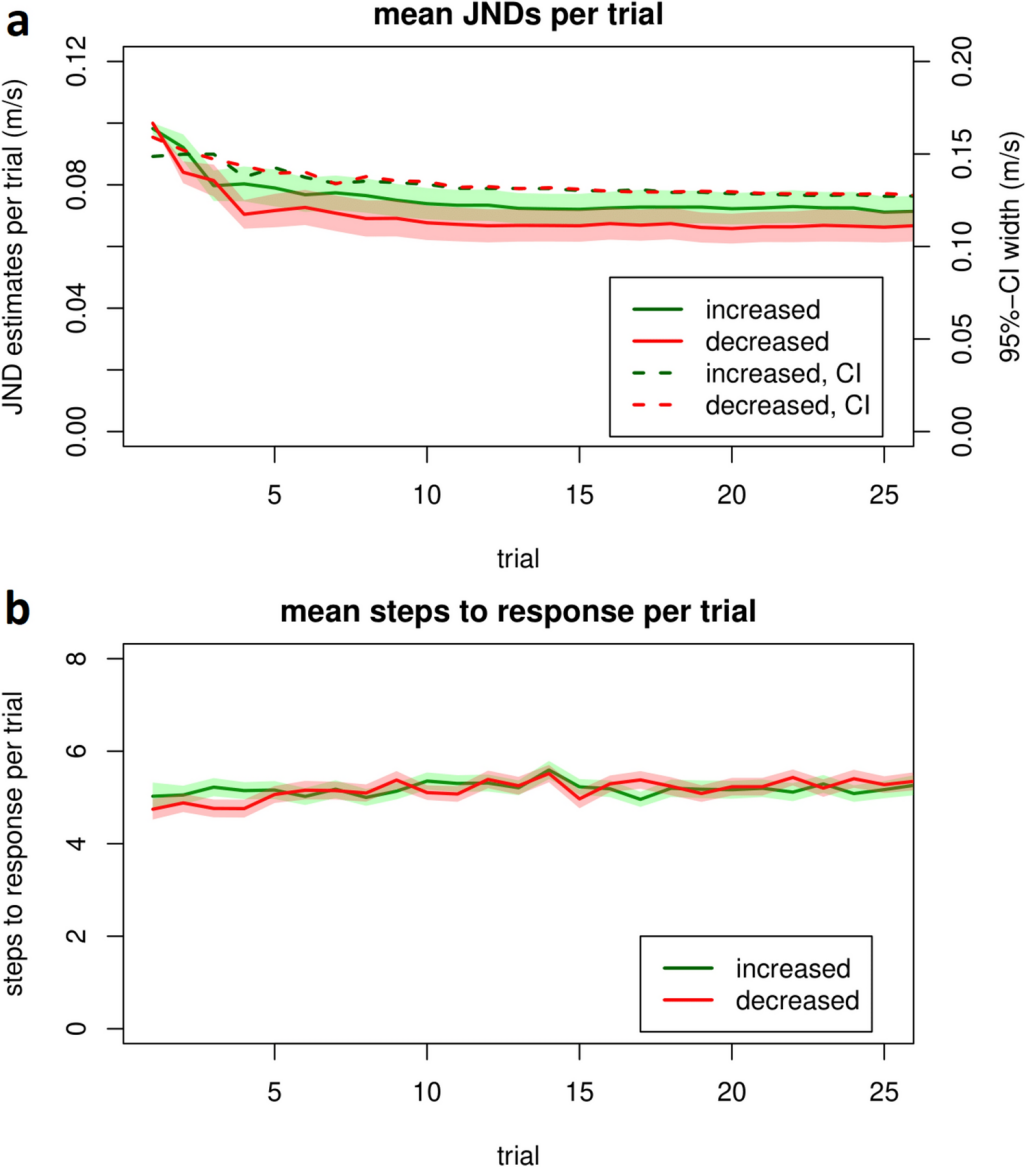
Trajectories of threshold estimates and mean steps to response. (**a**): Mean trajectories of the absolute threshold estimates for increased-speed perturbations (green) and decreased-speed perturbations (red) for each trial per block. The x axes end with the minimum number of trials presented to any participant in any block (this number could vary as we fixed the time and not number of perturbations per block). Shaded areas indicate ± 1 between-participant SEM. Threshold estimates started at 10% and rapidly and roughly asymptotically approached range of the final JND for both perturbation types. Dashed lines show the mean width of the 95%-confidence intervals of the estimates for each trial, computed from the QUEST’s probability density function. (**b**): Mean *steps to response* for each trial, depending on the perturbation type. Trajectories did not differ between increases (green) and decreases (red).

To further investigate possible differences in the detection of speed increases and decreases, we looked at how quickly the respective thresholds were reached. Figure 4a shows the mean trajectories of the absolute threshold estimates for increases and decreases. Both approach the threshold very quickly and then show asymptotic behavior. SEM (shaded areas; ± 1 SEM) of increases and decreases overlap over the whole trajectories, indicating again no difference between types. We also plotted the mean width of the 95% confidence interval of the probability density function for each trial (dashed lines), which quantify the mean precision of the estimate (in contrast to the shaded error bars, which quantify between-participant variability and this the precision of the mean estimate). These widths showed a similar asymptotic behavior as the threshold estimates, also with no difference between perturbation types.

Next, we investigated the average number of steps per trial needed to give a response (“*steps to response”*). Overall, participants responded on average after 5.11 ± 1.8 steps. Splitting up by perturbation type, no difference was found between increases (5.12 ± 1.8) and decreases (5.11 ± 1.8), (*t*(47) = -0.04,* p* = 0.969) with a corresponding Bayes factor of BF_10_ = 0.16. In line with these findings, trajectories again did not differ by type and both showed an asymptotic pattern (Fig. 4b). We then investigated whether there was an overall relationship between response speed and threshold – whether participants who responded quicker were better or worse at detection than those that responded more slowly. To avoid individual differences in how quickly participants got accustomed to the task and maximize the chance of getting trials close to threshold (so the subjective difficulty should be comparable), we looked specifically at trials 16 to 25. These were chosen because no participant completed fewer than 25 trials in any block.

The overall correlations of mean *steps to response* and mean JNDs over these trials, calculated per participant and perturbation type was *r* = -0.28 (*t*(94) = − 2.86,* p* = 0.005, BF_10_ = 9.48), and for speed increases *r* = -0.36 (*t*(46) = -2.58,* p* = 0.013, BF_10_ = 5.13) and decreases *r* = -0.23 (*t*(46) = -1.58,* p* = 0.121, BF_10_ = 0.95). Thus, participants who took longer with their responses were descriptively somewhat better at detecting the perturbations, perhaps indicating different response strategies, but the data pattern was not conclusive.

## Discussion

Here, we measured the perception threshold of inter-leg speed differences for increased-speed and decreased-speed perturbations while walking. We provide precise JNDs for speed differences, measured with an adaptive procedure on a large sample. Interestingly, we found no differences in average JNDs between increases and decreases, while our results indicate a considerable variability between *participants* for both perturbation types. Further, we show that the reliability of the JND estimates within each perturbation type was comparable to the correlation between JNDs for each type, suggesting that similar cues are used for each.

Knowing when participants are aware of perturbations can be of interest for a variety of reasons: To know when they might apply explicit strategies to adjust the corresponding motor actions, to better understand self-motion perception by understanding when humans notice that it is externally manipulated, or to know whether an experimental manipulation will work. For this, robust estimates of perception thresholds are fundamental. Our average threshold estimates for speed increases and decreases are somewhat lower (< 8%) than in previous findings^16,17^. However, in contrast to studies that perturbing one full stride cycle or a single stance phase^17,18^, we induced perturbations over multiple steps, thereby placing less emphasis on responding quickly to instead focus only on accuracy, and finding some participants’ individual thresholds seem to be even much lower, as we show for the first time in a large sample. In addition to testing more participants than is typically the case, we also had the advantage of using an adaptive threshold measurement, the QUEST procedure, as it enables individual threshold estimations for a wide range and higher resolution compared to constant stimuli presentation or methods of limits^16^. This has several advantages: Motivation is optimized, ground and ceiling effects are avoided, trials far from thresholds are taken less into account (e.g. difficulties in understanding instructions or response mappings) and particularly, on-the-fly measurements provide a large number of estimates near the actual threshold. In fact, when analyzing our data, we found that we could have likely made even more optimal use of this method, as asymptotic behavior of both the estimates and the estimate precision (Fig. 4a) was visible after as little as 15 trials. Consequently, the measured JNDs were relatively stable, as our reliability estimates (provided by the correlations between consecutive blocks of the same perturbation type) show. It will be interesting to investigate what factors may be behind these strong inter-individual differences in perturbation detection. Starting points for future research could for example be bodily self-awareness, footwear or experience in treadmill walking^32^.

That said, there are some limitations of our study. One is related to the instruction of the question to be answered. As we were interested in the detection of the perturbation, we used a 2AFC task asking “left or right faster” (instead of “which belt is manipulated”). However, this could lead to an asymmetry in responses, as for example a decelerating perturbation is induced on the left side, but the correct response in this case is “right belt running faster”. It may also lead participants to focus on speed differences rather than immediate biomechanical consequences of an acceleration or deceleration. It can also be confusing to participants if they are close to threshold and hardly able to distinguish the perception of “left belt running faster” and “right belt running faster”. To account for this, a possible approach may be to assess the perturbation detection by using a physiological marker such as pupillometry.

We also compared in-depth the detection performances of increased speed and decreased speed, respectively. It is plausible to expect differences here, because biomechanical dynamics, receptors and information differ^33^ and could lead to a different perception of the same speed differences. Interestingly, we found no difference in the mean perception threshold for speed perturbations between increases and decreases, despite the physiological differences between perturbation types and for both, our results indicate that participants could detect even small speed differences. Further, individuals’ JNDs for the two perturbation types were highly correlated, to an extent as one would expect from the respective reliabilities with two perfectly correlated constructs, and the majority of variability was between participants, not perturbation types. This means that participants who are good at detecting one perturbation also tend to be good at detecting the other perturbation and suggests that detecting speed differences is based on similar mechanics for speed increases and decreases.

As expected, participants rapidly reached an asymptote while approaching their individual threshold. Here, too, we found similar trajectories for both perturbation types when looking at the mean trial-wise threshold estimates (Fig. 4a). Finally, we investigated whether response strategies may be behind the large observed variability between participants. A potential explanation might be that participants with smaller JNDs may use more steps for collecting more information about the perturbation before giving a (then more thorough) response. We calculated the correlation of the mean *steps to response* and the JND of each participant, finding – though only descriptively for speed decreases – the suspected negative trend overall and for increases as well as decreases, indicating that more steps were associated with lower JNDs.

In our experiment, we focused on the precise measurement of individual JNDs. Certain related measures or experimental variations may also be of interest, but beyond the scope of this particular study. First, perturbations lasted a maximum of only 10 steps, so we can exclude adaptation effects, as most split-belt adaptation paradigms last about 2 min^16^. Within 10 steps, one might look at fast adjustments in step length asymmetry or carefully at motor aftereffects, but it may not be feasible to distinguish fast and slow adaptation components and analyze gait patterns^8,34,35^. However, even short-term adjustments are difficult to quantify since we used an adaptive procedure to determine the strength of the perturbations, meaning that each participant was exposed to a different set of perturbations. Second, in contrast to previous gait studies^9,18^, we did not use a self-selected walking speed but a fixed baseline speed of 1 m/s for our QUEST procedure. While this speed has been proven to be a comfortable speed to induce motor perturbations while participants performed another task in previous studies using the same setup^36^, one might conceivably obtain different results with self-selected speeds^37^, or by applying normalization procedures based on leg length or stability^38^, which could be interesting to investigate. Third, generalizability is an important issue as walking behavior depends on a large range of parameters and their interactions^8,37,39^, only some of which can be manipulated here. For example, using young and healthy adults likely affected our results^16,19^, as did the choice of the baseline speed^16,18^. Environmental setups often varied between experiments, for example with visual environments sometimes reduced^9^ or even largely missing^18^, so we aimed for a more ecologically valid set of parameters using this dynamic environment that might improve detection performance by providing also visual information to make walking more realistic. Comparing different environments and manipulating visual information might also reveal differences in thresholds^16^.

Taken together, we report precise estimates or speed-difference thresholds in split-belt walking for increased-speed and decreased-speed perturbations in young and healthy participants. We show that individual threshold for speed increases and decreases are comparable within participants and variability is mainly found between participants. These results emphasize the importance of considering individual differences while investigating perturbation detection and potentially explicit sensorimotor adaptation, and thus provide implication for a variety of research in self-motion perception, sensorimotor adaptation, and fall prevention.

## Declaration

## Consent to participate

After being fully informed about the study, participants consented in writing to participate prior to the experiment.

## Consent for publication

Participants consented in writing for their data to be made publicly available prior to the experiment.

## Data Availability

Merged data for all experiments are available at https://doi.org/10.17605/OSF.IO/B7K82.
